# Cholera in Africa: Current trends, challenges, and control towards elimination strategies

**DOI:** 10.1371/journal.pntd.0014325

**Published:** 2026-05-28

**Authors:** Claude Mambo Muvunyi, Emmanuel Edwar Siddig, Eman Taha Osman Ali, Patrick Gad Iradukunda, Pierre Gashema, Jean de Dieu Harelimana, Mazyanga Lucy Mazaba, Jean Kaseya

**Affiliations:** 1 Rwanda Biomedical Center, Kigali, Rwanda; 2 Pan Africa Biomedical Institute, Kigali, Rwanda; 3 Faculty of Medical Laboratory Sciences, University of Khartoum, Khartoum, Sudan; 4 Rwanda Food and Drugs Authority, Kigali, Rwanda; 5 African Medicines Harmonization Initiative, AUDA NEPAD, Johannesburg, South Africa; 6 Africa Centres for Disease Control and Prevention (Africa CDC), Eastern Regional Coordinating Centre, Nairobi, Kenya; 7 Africa Centres for Disease Control and Prevention (Africa CDC), Addis Ababa, Ethiopia; Centers for Disease Control and Prevention, UNITED STATES OF AMERICA

## Abstract

Cholera remains a substantial public health challenge in Africa, despite the availability of effective vaccines, treatment, and preventive measures. Systemic issues such as inadequate water, sanitation, and hygiene (WASH) infrastructure, weak disease surveillance, socio-economic disparities, and ongoing conflict and instability drive its persistence. This article provides the current situation of cholera in Africa, highlighting systemic barriers to control, including data deficiencies, limited diagnostic capacity, and infrastructural deficits. Crucially, we underline the importance of integrated strategies encompassing enhanced surveillance, targeted vaccination campaigns, WASH improvements, and addressing social determinants like poverty and displacement. Strengthening multisectoral and multidisciplinary collaborations and leveraging technological innovations are vital steps toward eliminating cholera in Africa. Achieving this goal requires sustained political commitment, increased resource mobilization, and context-specific solutions tailored to the diverse challenges faced by vulnerable communities.

## Introduction

Cholera, a highly preventable and treatable waterborne disease caused by *Vibrio cholerae*, continues to devastate African populations despite significant scientific advances and effective intervention tools [1]. Its persistence illuminates deep-seated health inequities, systemic infrastructure failures, and social neglect in vulnerable populations [1,2]. While some countries have made notable progress in cholera control, many African countries remain plagued by recurrent outbreaks, high mortality rates, and weak response systems [3]. Between 2014 and 2024, reported cholera cases surged from 105,287 cases and 1,882 deaths across 19 countries to 254,075 cases and 4,725 deaths across 20 countries—representing a 141% increase in cases and a 151% increase in mortality [1–3]. This ongoing crisis underscores the urgent need for comprehensive, multisectoral strategies that address both immediate health threats and the root social, economic, and infrastructural determinants fueling persistent transmission [1–3]. The integration of advanced water monitoring technologies, including molecular surveillance, plays a vital role in early outbreak detection and targeted interventions [4]. This article highlights the current cholera landscape in Africa, examines systemic barriers impeding control efforts, and advocates for a holistic, innovative, and context-specific response to eliminate cholera by 2030. We emphasize integrated strategies such as enhanced surveillance, targeted vaccination, WASH improvements, and tackling poverty and displacement.

## Current cholera situation in Africa compared with other regions

**Fig 1:**
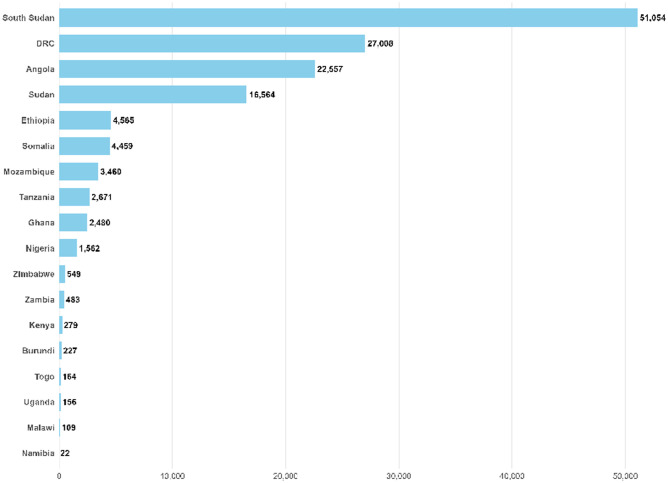
Number of cholera cases as from January up to 22 May 2025 (adopted based only on data from World health organizations, the figure was made by the authors from the extracted data).

**Fig 2:**
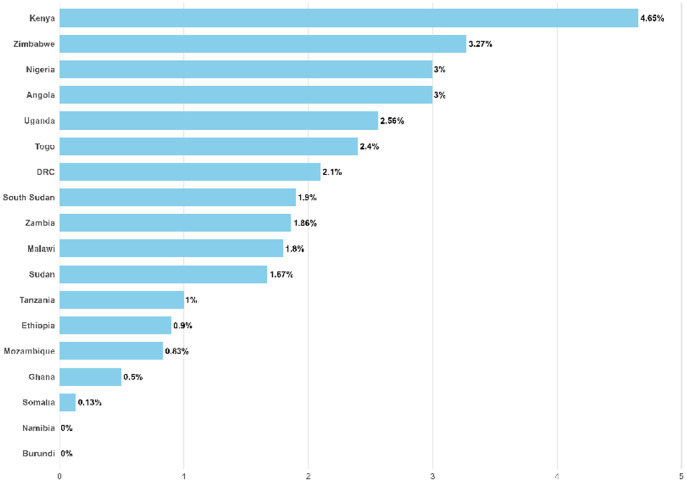
Case Fatality Rate of cholera cases as from January up to 22 May 2025 (adopted based only on data from World health organizations, the figure was made by the authors from the extracted data).

The persistent prevalence of cholera across Africa highlights a devastating gap between what is known about controlling this disease and the reality of its ongoing toll. Countries such as Angola, South Sudan, and DRC continue to report thousands of cases and significant mortality, reflecting systemic failures in public health infrastructure, sanitation, and disease surveillance [5]. As of 22 May 2025, Angola has reported 22,557 cases with a case fatality rate of 3%, and South Sudan’s numbers soar to over 51,000 cases with nearly 1,000 deaths—equating to a case fatality rate of 1.9% (Figs 1 and 2). These figures are alarming, yet they likely underestimate the true scale due to underreporting and limited testing capacity. Reportedly low cholera case numbers in many countries on the African continent may offer a misleading picture, especially when neighboring countries are battling major outbreaks. In many cases, weak surveillance infrastructure, delayed outbreak recognition, and limited diagnostic capacity can obscure the true scale of transmission and undermine timely response. It impairs rapid response efforts and hampers resource allocation. The lack of accurate data means that many outbreaks go unnoticed until they escalate into full-blown crises, costing lives and resources that could be saved with timely intervention.

## The root causes and barriers to control

### Barriers to effective cholera prevention

The continued widespread transmission of cholera across Africa can be attributed to a complex nexus of interconnected social, economic, infrastructural, environmental, and political factors [3,6]. At the core is widespread poverty, which severely limits access to safe drinking water and adequate sanitation facilities, thereby creating conditions conducive to cholera transmission. Overcrowded informal settlements and rural communities often lack basic infrastructure, such as clean water sources and waste disposal systems, which perpetuate fecal-oral transmission pathways [7].

One major barrier to effective prevention is the accurate estimation of cholera cases [7]. Inaccurate reporting plays a crucial role in sustaining ongoing transmission and hampers response efforts. Inaccurate reporting significantly contributes to the continued transmission of cholera and undermines response efforts. For example, a study by Ali and colleagues revealed that the WHO reported 331,337 cholera cases between 2008 and 2012, which actually constituted just 11.6% of the estimated total cases [8]. Moreover, the reported 6,335 deaths accounted for a mere 6.6% of the estimated fatalities during that period [8]. Moreover, research conducted by O-Tipo Shikanga and colleagues during a 2008 cholera outbreak in western Kenya uncovered that the actual number of cases was 46% higher and the fatalities were 200% greater than those officially documented, highlighting the effectiveness of active community case-finding in uncovering the true extent of the outbreak. These findings illustrate the critical need for accurate data to effectively manage cholera outbreaks and implement appropriate response strategies [9]. This inadequacy affects strategic planning and results in improper resource allocation for cholera risk reduction and prevention, contributing to recurrent outbreaks and an increasing public health burden [10].

Conflict and political instability further exacerbate the cholera situation by displacing populations into overcrowded, unsanitary camps, disrupting health services, and destroying existing water and sanitation infrastructure [3,11]. Notably, more than half (12) of the 22 countries classified by the World Bank as fragile and conflict-affected in 2025 are in Africa [12]. Addressing conflict-triggered cholera epidemics requires a multifaceted approach that includes strengthening peacekeeping efforts and promoting stability in conflict-affected regions. Collaborating with humanitarian organizations to provide immediate health services, re-establishing water and sanitation infrastructure, and ensuring safe access to healthcare facilities are critical to combating cholera in these settings. Moreover, engaging local communities in decision-making processes can enhance resilience and foster a sense of ownership over health interventions, thereby mitigating the impact of conflict on cholera outbreaks.

Environmental factors such as climate variability and climate change influence the proliferation and spread of *Vibrio cholerae*, with floods and droughts increasing the risk and geographic extent of outbreaks, a case of Sudan and South Sudan, for example. Recurrent flooding in South Sudan and prolonged droughts in Sudan have damaged water infrastructure, contaminated surface water sources, and displaced populations into overcrowded camps, conditions that create ideal environments for cholera transmission [13]. In particular, rising temperatures, altered rainfall patterns, and extreme weather events such as floods and droughts are increasingly linked to cholera outbreaks, a recent case of Sudan and South Sudan. These climate-induced shifts affect water availability and quality, disrupt sanitation infrastructure, and expand the geographic range of transmission, especially in low-resource settings where adaptive capacity is limited.

### Challenges in timely control of cholera outbreaks

Defects in the Water, Sanitation, and hygiene (WASH) infrastructure and practices act as the primary barrier to controlling cholera [14]. Many African countries face challenges in delivering sufficient sewage treatment and waste management, resulting in the pollution of drinking water sources. The prevalent inadequacy of proper sewer treatment facilities across many African countries hinders effective wastewater management. For instance, in Addis Ababa the capital of Ethiopia, treatment facilities process less than 3% of wastewater [14]. Likewise, in Kenya especially at Kisumu district, failures at pump stations cause sewage to overflow at upstream access points, leading to untreated sewage being discharged directly into Lake Victoria [14].

Additionally, the fragility of health systems across much of Africa significantly hampers effective cholera control and response efforts [3]. Many countries grapple with limited diagnostic capacity, which compromises the ability to quickly and accurately identify cholera cases in the field. This challenge is compounded by inadequate disease surveillance systems, essential for timely detection of outbreaks and coordination of response activities. A shortage of trained healthcare and surveillance personnel in endemic regions further impairs prompt identification, reporting, and response to cholera cases. Moreover, the general absence of molecular surveillance technologies—crucial for tracking specific pathogen strains, understanding transmission pathways, and detecting antimicrobial resistance patterns—restricts efforts to manage outbreak dynamics effectively. Unfortunately, due to substantial resource constraints, these advanced technologies are rarely accessible in the areas most severely affected by cholera.

According to estimates from the World Health Organization, Africa’s overall healthcare index stands at only 0.32 on a scale where 1 represents optimal healthcare access. This alarming figure indicates that, on average, the continent’s health systems can provide just 32% access to essential healthcare services. In some nations, such as the Central African Republic, the healthcare index drops to an alarming low of 0.12, illustrating severe deficiencies in healthcare infrastructure and capacity [15]. Such limited access means that many individuals suspected of having cholera never reach healthcare facilities, leading to significant underreporting and gaps in surveillance data, creating a substantial blind spot in outbreak detection and management. The challenge is intensified by a general lack of public awareness regarding cholera surveillance efforts [16], reducing community engagement and cooperation. Misinformation, stigma surrounding cholera and related illnesses [17], and deep-seated mistrust of local health authorities often result in cases—and even deaths—going unreported or concealed. This widespread concealment hampers early detection, impedes rapid response initiatives, and allows cholera transmission to continue unmitigated in many affected communities. Despite the existence of proven interventions, such as oral rehydration therapy, oral cholera vaccines, and sanitation improvements, their deployment frequently faces logistical challenges, limited funding, and insufficient political commitment. Additionally, behavioral and cultural factors influence community engagement, with misinformation and mistrust obstructing participation in prevention activities [17]. Interestingly, in a study conducted by Patel and associates identified key elements of a resilient community, including the availability of community resources, effective communication, local knowledge, training, and education. Thus, the resilience of households and communities is vital for preventing disease outbreaks, including cholera. Resilient households and communities can resist, absorb, and recover from the risks and impacts of cholera outbreaks in a timely and efficient manner, thereby preventing recurrent outbreaks. Unfortunately, this resilience is lacking in many African communities [7,18]. For example, while handwashing with soap is a critical intervention to prevent cholera transmission, numerous households in Africa lack access to drinking water, let alone water for handwashing. Even when water is available, households may be unable to afford soap. Additionally, a deficiency in local knowledge and ineffective communication about cholera risks further heightens the risk of infection, leading to undetected outbreaks and inadequate prevention at both household and community levels. While these challenges extend beyond cholera, they are also crucial for preventing other diseases, including COVID-19 and Ebola Virus Disease (EVD). Patel et al. also emphasized health—encompassing the pre-existing health status and access to healthcare services before, during, and after emergency events—as a critical component of a resilient community [18]. The continent’s insufficient access to healthcare services exacerbates this challenge.

### Moving forward: A call for urgent, coordinated action

To eliminate cholera in Africa by 2030, it is crucial for nations to embrace the WHO’s cholera roadmap, titled “Ending Cholera – A Global Roadmap to 2030.” This initiative represents a collaborative effort involving cholera-affected countries, stakeholders, technical partners, and donors. The roadmap’s primary objective is to achieve a 90% reduction in cholera mortality rates and eliminate disease transmission across as many nations as possible by the target year of 2030 [19,20]. The following strategies outline key actions for achieving this goal:

1. Effective Implementation of the Global Cholera Roadmap and Strategies

This includes enforcing public health legislation to establish regulations that facilitate cholera prevention efforts. Strengthening health systems and enhancing the capacity to respond to outbreaks will be critical for effective implementation.

2 Risk Communication and Community Engagement

Public awareness campaigns need to educate communities about cholera health risks and preventative measures. These campaigns should leverage trust in local leaders and health workers to enhance community participation in prevention activities, thereby fostering a proactive approach to health management.

3 Improving Water, Sanitation, and Hygiene (WASH)

Short-term measures should focus on providing immediate access to safe drinking water and sanitation facilities in cholera-endemic areas. Long-term strategies should include substantial investments in infrastructure improvements, such as sewage treatment systems and hygiene education campaigns. Establishing routine monitoring and inspection of facilities will ensure consistent adherence to hygiene standards.

4 Strengthening Surveillance and Data Management

Establishing robust disease surveillance systems for real-time reporting and data sharing is essential. Incorporating innovative digital technologies—such as mobile health applications and AI-driven data analytics—will enhance outbreak detection, facilitate timely responses, and improve overall data quality.

5 Scaling Up Vaccination

Mass vaccination campaigns targeting high-risk zones should be prioritized. Prequalified vaccines like Dukoral, Sanchol, and Euvichol should be widely distributed with a focus on vulnerable populations to reduce transmission rates effectively [20,1].

6 Enhancing Case Management Strategies

Training healthcare providers on recognizing cholera symptoms and swiftly implementing appropriate treatments is vital. Emphasis should be placed on effective hydration strategies and the provision of essential supplies at clinics to manage outbreaks efficiently.

7 High-Level Political Commitment, Multisectoral Coordination, and Financing

Strong political will is necessary to prioritize cholera prevention as a public health imperative. Governments should collaborate with international organizations, NGOs, and private sector partners to mobilize resources, ensuring adequate funding for cholera prevention and control initiatives. Establishing multisectoral coordination mechanisms will streamline efforts and facilitate knowledge sharing among stakeholders.

### Conclusion

Cholera in Africa remains a critical public health challenge deeply rooted in systemic inequities, socio-economic disparities, and deficiencies in water and sanitation infrastructure. Despite proven interventions such as vaccines and improved disease surveillance, ongoing outbreaks highlight significant gaps in implementation and political commitment. To effectively combat cholera, African governments and policymakers must adopt a coordinated, multisectoral approach. Key strategies include enhancing surveillance systems, prioritizing Water, Sanitation, and Hygiene (WASH) initiatives, scaling up vaccination efforts, and fostering community engagement. Additionally, strong political commitment and resource mobilization are crucial to prioritizing cholera control as a public health imperative.
